# Supplementary figures and images for: Phyllosphere microbial communities are modulated by pathogen coinfection, but not a plant defense hormone
Source: PLoS One. 2026 Jan 29;21(1):e0341614. doi: 10.1371/journal.pone.0341614 (PMC12854442; doi:10.1371/journal.pone.0341614)

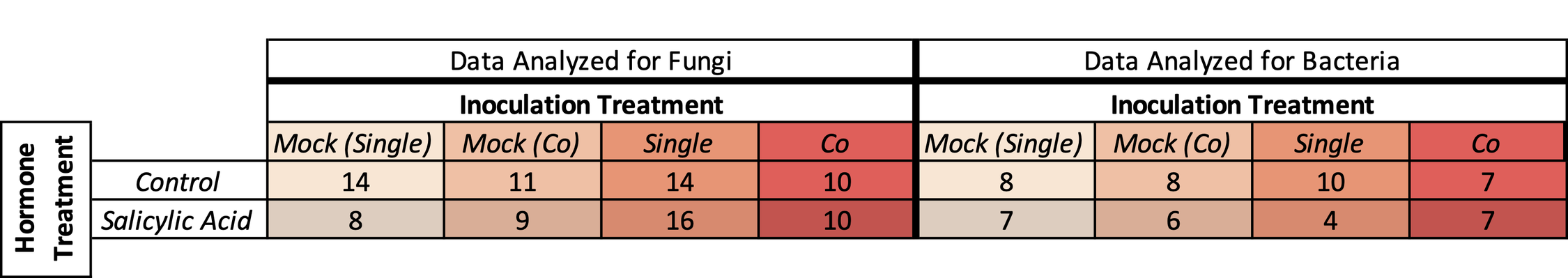

Supplement: S1 Table — In total, 92 samples were analyzed for fungal diversity, community composition and abundance, and 57 samples were analyzed for metrics of bacterial community structure. Table abbreviations are as follows: “Mock (Single)” = plants that received a mock inoculation of R. solani; “Mock (Co)” = plants that received a mock co-inoculation of R. solani and C. cereale; “Single” = plants that received an inoculation of R. solani; “Co” = plants that received a co-inoculation of R. solani and C. cereale. Plants across inoculation treatment groups either received a hormone treatment of sterile water (control), or salicylic acid. (TIF) [file pone.0341614.s001.tif]

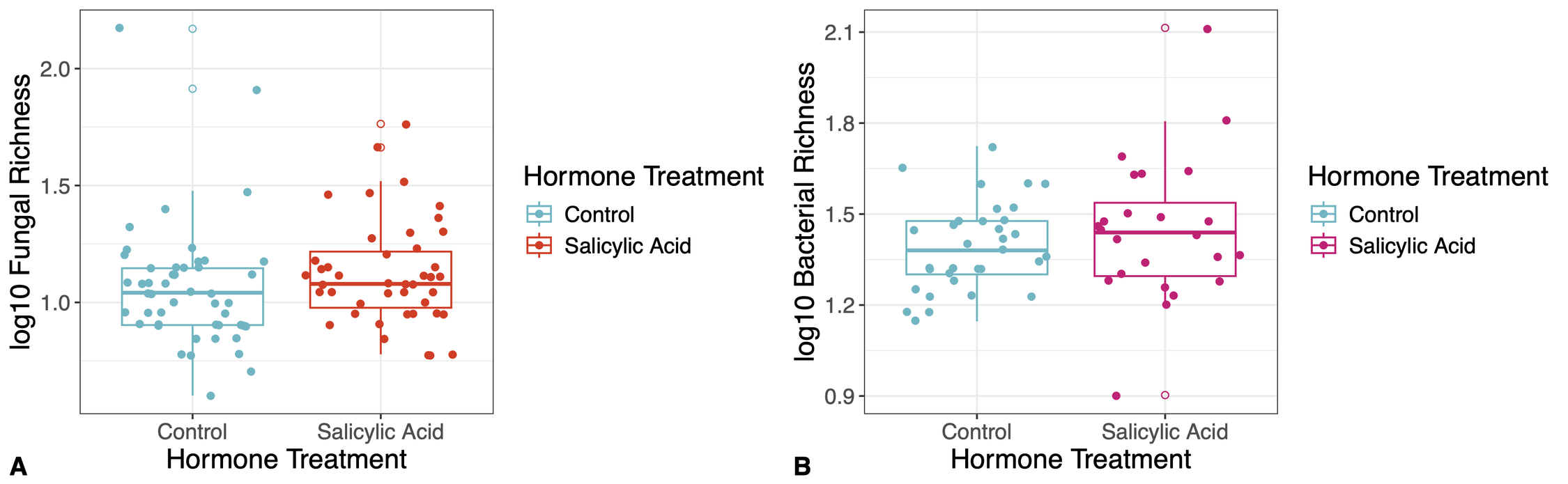

Supplement: S1 Fig — Panels show (A) the application of salicylic acid did not influence the richness of fungi on plant leaves (p = 0.22) and (B) the application of salicylic acid did not influence the richness of bacteria on plant leaves (p = 0.29). (TIF) [file pone.0341614.s002.tif]

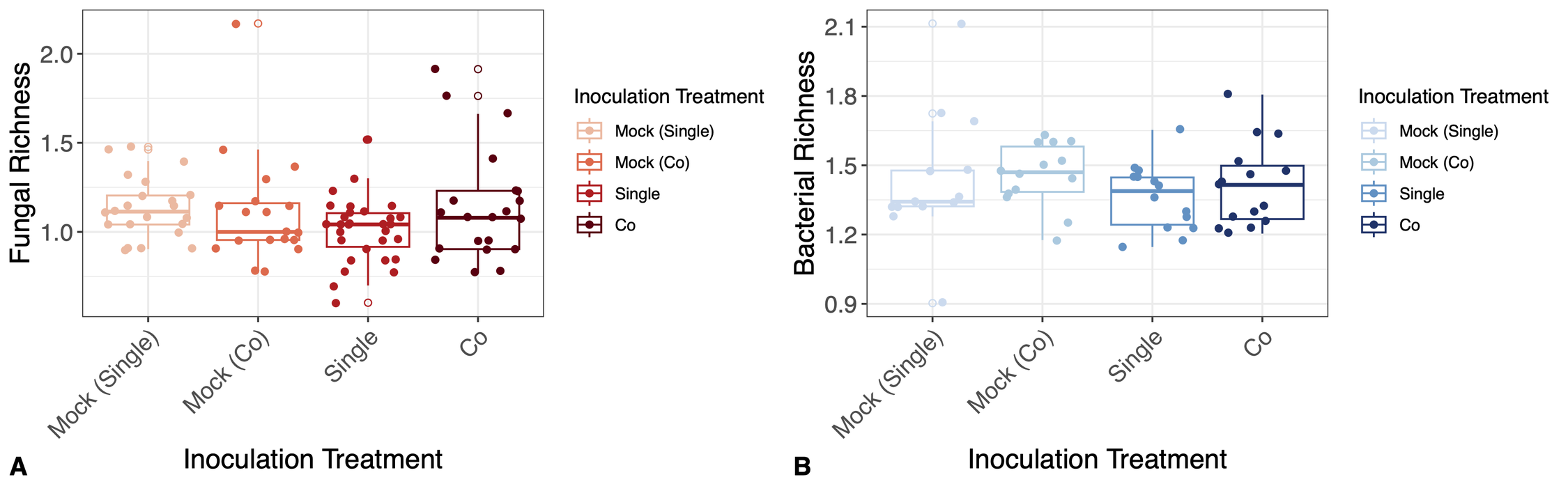

Supplement: S2 Fig — Panels show (A) inoculation treatment did not influence fungal richness (p = 0.14) or (B) inoculation treatment did not influence bacterial richness (p = 0.55). (TIF) [file pone.0341614.s003.tif]
