# Supplementary material for: Phyllosphere microbial communities are modulated by pathogen coinfection, but not a plant defense hormone
Source: PLoS One. 2026 Jan 29;21(1):e0341614. doi: 10.1371/journal.pone.0341614 (PMC12854442; doi:10.1371/journal.pone.0341614)
Supplement: Attachment — Submitted filename: Response to Reviewer #1.docx [file pone.0341614.s006.docx]

Response to Reviewer #1

Reviewer #1

The purpose of this study was to examine how sequential exposure to salicylic acid and a foliar fungal pathogen would influence the diversity, community structure, and differential abundance of both the fungal and bacterial communities of tall fescue. In general, the investigated treatments influenced neither the bacterial or fungal community structure nor differential abundance. The initial conclusion of the discussion is not well supported by the results. Fungal community composition variation was low in the co-inoculated (line 400-404); not “significant changes” as stated in the discussion.

***Author:*** *Thank you for taking the time to provide thoughtful and constructive feedback on our work. Your suggestions will help us improve the clarity of and strengthen our manuscript. We appreciate the reviewer’s concern to make sure that the manuscript’s conclusions are supported by the results. That said, we disagree with that “the initial conclusion of the discussion is not well supported by the results”. The finding that fungal community composition was affected by pathogen inoculation was supported by a standard and robust statistical test (PERMANOVA, F*_3,87_ = 1.60, p = 0.021*). However, the reviewer is correct that while we observed statistically significant differences in fungal community composition between treatments, the effect size was small (R² = 0.052). We have revised the discussion to highlight that while this treatment effect was statistically significant, it explained a small proportion of the variation in fungal community composition (lines 437-438 (track changes manuscript lines 448-449)); lines 440-443 (track changes manuscript lines 451-454)). We also updated the results section to explicitly note both the significant effects and the small effect size within the same sentence (lines 404-406 (track changes manuscript lines 415-417)).*

Whether fungi and bacteria interact with each other was not directly examined by this study and such should be removed from the discussion.

***Author:*** *The reviewer makes a valid point. Our study design was not specifically structured to test direct fungal-bacterial interactions. We have revised the discussion to clarify that our observations suggest differential responses of fungal and bacterial communities to the same treatments, rather than speculating about fungal-bacterial interactions (deleted text starting at line 446 (track changes manuscript line 457).*

The authors suggest that their fungal pathogens create necrotic lesions which would be favored by decomposer fungi and yet, most inoculated plants in this study remained asymptomatic.

***Author:*** *This is a valid point. The manuscript addressed it in the Discussion, suggesting effects of asymptomatic infection as a topic for future research (lines 481-486 (tracked changes manuscript lines 522-527)). That said, we acknowledge that our low symptomatic infection rates (approximately 30% for R. solani and 10% for C. cereale) limit our ability to connect necrotic lesion formation to the observed fungal community changes. We expanded the Discussion to further acknowledge this limitation and suggest that the observed effects may be due to either: (1) asymptomatic infections that still alter the within-leaf environment even without visible symptoms, or (2) localized tissue changes not visible at our assessment scale (lines 446-448 (tracked changes manuscript lines 457-459)).*

As for the findings from the salicylic acid treatment, the authors should have a better rationale for their chosen concentration. It’s possible that their concentration wasn’t biologically relevant to their host.

***Author:*** *We agree that selecting biologically relevant treatments is an important part of the methods for any experiment, and we considered our selection of salicylic acid concentration thoroughly before conducting our experiment. Our manuscript addressed this topic in a full paragraph in the Discussion, but this reviewer comment indicates to us that we need to also address it in the Methods. The Discussion explained that we selected 0.72 mM salicylic acid based on previous literature showing effectiveness in this concentration range (0.1-8.0 mM) across diverse host species, and more specifically our concentration falls within the range that has demonstrated biological activity in other grass species (lines 472-475 (tracked changes manuscript lines 509-516)). The Discussion also acknowledged that the optimal concentration may be species-specific for our grass host (lines 465-467 (tracked changes manuscript lines 502-504)). Additionally, the Discussion suggested that future studies could examine differing host responses to foliar vs. systemic alteration of defense hormones (lines 490-493 (tracked changes manuscript lines 531-534)). To address the reviewer concern, we added text to our methods section to also include the underlying rationale for our decision to treat plans with 0.72 mM salicylic acid (lines 140-143; lines 147-150 (tracked changes manuscript lines 140-143; lines 147-150)).*
