# Supplementary material for: Phyllosphere microbial communities are modulated by pathogen coinfection, but not a plant defense hormone
Source: PLoS One. 2026 Jan 29;21(1):e0341614. doi: 10.1371/journal.pone.0341614 (PMC12854442; doi:10.1371/journal.pone.0341614)
Supplement: Attachment — Submitted filename: PONE-D-25-22997_R1_Review report.docx [file pone.0341614.s005.docx]

The manuscript titled “Phyllosphere microbial communities are modulated by pathogen coinfection, but not a plant defense hormone” presents interesting findings showing that co-infection by *Rhizoctonia solani* and *Colletotrichum cereale* significantly altered and enhanced the diversity of phyllosphere fungal communities in tall fescue, whereas exogenous application of salicylic acid had no significant effect on either fungal or bacterial communities. These results highlight that pathogen interactions play a more dominant role than salicylic acid in shaping phyllosphere microbiomes. Overall, the manuscript is well written and provides valuable insights to the scientific community. However, I recommend several revisions and clarifications before the manuscript can be considered suitable for publication.

Line 20-38 Your abstract lacks a clearly defined problem statement and a well-articulated research gap that directly connects to the objectives of your study. In particular, lines 20–24 should emphasize the novelty of your work and how it addresses an existing gap in current knowledge. The description of treatments involving salicylic acid and plant pathogens (lines 25–27) is somewhat unclear and requires clarification. It is also important to specify the concentration of salicylic acid used in this study. Additionally, please clarify whether you used only a sole inoculation with *Rhizoctonia solani* or if *Colletotrichum cereale* was also used in sole inoculation, along with the co-inoculation treatment involving both pathogens. If only *R. solani* was used for sole inoculation, please justify the rationale for selecting it over *C. cereale*. In the results section (lines 27–35), the logical flow of presentation needs improvement to support a stronger and more cohesive conclusion. Some statements are also ambiguous — for example, in line 31, the phrase “compared with *R. solani*, co-inoculation with *C. cereale*” appears technically and grammatically inconsistent, as it implies co-inoculation only with *C. cereale*. Similarly, lines 32–33 are unclear regarding whether sole or co-inoculation had no significant effect on bacterial communities. Furthermore, the concluding statements of your abstract appear contradictory. You mention that co-inoculation had a stronger influence on fungal communities, yet in the following sentence, you generalize the effect to all microbial communities, despite your results indicating no significant impact on bacterial communities. This inconsistency also extends to the manuscript title, which refers to “phyllosphere microbial communities,” while the results primarily highlight effects on fungal communities. Lastly, ensure consistency in terminology—use either “co-infection” or “co-inoculation” throughout the manuscript. It would also strengthen your abstract to include a concise statement on the future research directions or potential implications of your findings, highlighting how they could advance understanding in this field.

Line 38-39 Your manuscript currently lacks a list of keywords relevant to your study. Most journals require keywords to be included immediately after the abstract and before the introduction section. Including well-chosen keywords is important, as they enhance the discoverability and global accessibility of your work within the scientific community. Please add appropriate keywords that accurately reflect the core themes, experimental focus, and major findings of your study.

Line 40-88 Your introduction lacks a clear and logical flow between statements and paragraphs. For example, in the first paragraph, the discussion shifts from salicylic acid (SA) to general phytohormones and then returns to salicylic acid, which disrupts the coherence of the narrative. To improve the flow, it is recommended to organize the content more systematically—begin by introducing the general role of phytohormones in plant defense mechanisms, and then gradually narrow the focus to salicylic acid and its specific relevance to your study. This progression will help readers follow the rationale of your argument more effectively. Additionally, if an abbreviation or acronym is not used later in the text, it should not be introduced, as this can create unnecessary complexity and reduce clarity. In the final paragraph (lines 76–79), instead of providing details about your experimental setup, it would be more appropriate to focus on developing a clear research gap derived from existing studies. This should highlight why your study is needed and what makes it novel. Based on this identified gap, you can then logically formulate your study objectives and hypotheses to align with the overall research framework. A well-structured introduction should first describe the existing problem or challenge and its impact on plants or microbial communities, followed by a concise summary of the current understanding in the field. It should then clearly define the research gap and lead to the formulation of specific objectives and hypotheses aimed at addressing that gap. Revising your introduction to follow this logical sequence will greatly enhance its clarity, coherence, and scientific strength.

Line 90-302 It is suggested to reorganize the content of your methodology section to improve clarity and logical flow. For instance, in lines 94–97, you describe the treatments involving salicylic acid and pathogen inoculation, while the details regarding their preparation and application are provided later in the text. This separation makes it difficult for readers to follow. To enhance readability, consider presenting the preparation and application details immediately after the treatment descriptions. Additionally, the concentration of salicylic acid is not mentioned at the point of first reference, which could lead to confusion. Please ensure that the concentration is clearly stated where salicylic acid treatment is first introduced. Moreover, clarification is needed regarding the limited quantities of C. cereale and the rationale for not using it as a sole inoculation treatment. If the pathogen was available in insufficient quantities, it is unclear why additional inoculum was not prepared for the experiment. Please explain the reasoning behind this decision. Similarly, provide justification for including a mock treatment for sole or co-inoculation when separate sole and co-inoculation treatments were already included. In lines 103–108, the discussion of Epichloë coenophiala seems unnecessary within the methodology. Instead, it would be more appropriate to address this background information in the introduction section to provide context for your experimental design. The seed sterilization protocol should also cite the original reference if it was adopted from previous studies; if it is a modified method, please explain why this particular approach was chosen. Furthermore, include complete details of the autoclaving conditions—temperature, pressure, and duration—used for soil sterilization. Also, please correct the spelling of “container” in lines 123 and 127. The information presented in lines 142–145 would fit better in the introduction section, where it can contribute to building background context. In addition, please specify the exact amount or volume of the 100 mg/L salicylic acid solution applied to each container or plant, and indicate whether it was a single or multiple application during the study period. The number of replications per treatment is also missing. Clarify why 59 plants were used for salicylic acid treatment while 57 plants were used for the control—were the replications unequal across treatments? The same for inoculation treatment in lines 168-171. Lastly, in lines 202–208, if the figure is not included within the text, you may remove the caption and footnote from the main body. Simply cite the figure number in the text and place the corresponding figure, caption, and footnote at the end of the manuscript or in accordance with the journal’s formatting requirements.

Line 304-445 It is recommended to make your section headings more concise and descriptive, clearly reflecting the content discussed under each. Avoid using sentence-like headings, as they can reduce clarity and make the manuscript appear less structured. Instead, use brief, focused heading that summarize the main idea or process covered in each section.

Line 447-506 Your discussion section would benefit from a clearer and more logical structure. It is recommended that you first present and interpret your main findings, and then discuss the underlying mechanisms that may explain these results. This should be followed by relevant supporting evidence from previous studies to strengthen your interpretations and demonstrate how your findings align with or differ from existing literature. Such an approach will create a more cohesive and scientifically sound discussion that effectively links your results to established knowledge. Additionally, your manuscript currently lacks a Conclusion section, which is an essential component in most journals. A well-written conclusion should succinctly summarize the key findings of your study in direct relation to your stated objectives and hypotheses. It should also highlight the main take-home messages, identify any limitations of the research, and suggest potential directions for future studies. Finally, discussing the practical implications and broader significance of your findings will help demonstrate the contribution of your work to the scientific community and its relevance to future research or practical applications.

**Final recommendations: Moderate revision**
