# Supplementary material for: Phyllosphere microbial communities are modulated by pathogen coinfection, but not a plant defense hormone
Source: PLoS One. 2026 Jan 29;21(1):e0341614. doi: 10.1371/journal.pone.0341614 (PMC12854442; doi:10.1371/journal.pone.0341614)
Supplement: Attachment — Submitted filename: Response to Reviewers.docx [file pone.0341614.s007.docx]

Response to Reviewers

*Dear Dr. Llorens,*

*Thank you for inviting us to revise our manuscript following our appeal. We thank the reviewers for their thoughtful and constructive feedback, which has substantially strengthened our manuscript.*

*In response to the comments from Reviewer 3, we have made several key improvements to the work. We have expanded the introduction to clarify the rationale for selecting* R. solani *and* C. cereale *as our focal pathogens, explaining that these species naturally co-occur and co-infect within the tall fescue system. We have also added text to further clarify the treatments in our factorial design, specifically the contents of the control hormone treatment. As suggested, we have added to our discussion by proposing several mechanisms for the shifts we see in fungal communities following coinfection. Lastly, we have acknowledged the limitations of our amplicon sequencing approach in the context of functional profiles. We believe these revisions have significantly improved the clarity and depth of the manuscript, and we appreciate the opportunity to address these important points.*

*In response to Reviewer 4, we have made substantial changes to our abstract, introduction, and discussion to improve narrative flow and readability.*

*We look forward to your evaluation of our revised submission and appreciate the opportunity to improve our manuscript through this process.*

Reviewer #3

In this study Geyer, Grunberg and Mitchell, investigated how application of hormone salicylic acid and coinfection by two fungal pathogens influenced fungal and bacterial communities on leaves of Lolium arundinaceum. I believe that the experimental design is well developed and the results are in line with the methods and objectives of the study. However, I have some concerns, which are outlined below.

*Author:* *Thank you for taking the time to provide thoughtful and constructive feedback on our work. We will address the concerns below.*

The authors do not clearly explain why they decided to use R. solani and C. cereale in their experiments. Is this co-infection possible in nature? Are they natural pathogens of L. arundinaceum?

*Author: This is a good point. We added information to the introduction (lines 80-81) explaining that* R. solani *and* C. cereale *are naturally co-occurring and co-infecting pathogens within the tall fescue system.*

What other defenses besides chemical defense does this plant have to prevent infection by pathogens?

*Author: Grass species have several physical defenses hindering colonization by plant pathogens, including possessing a waxy cuticle. In our study, we decided to focus on inducible and dynamic defenses which have known interactions with microbial communities, rather than physical defenses. However, we have added information to lines 41-42 to acknowledge that immune hormones are just one component of the multifaceted plant immune system.*

Beyond the taxonomic identity of the fungal and bacterial species found, what information do the authors have about their biology and interactions in the context of a bacterial community?

*Author: This is an important question that is likely dependent on ecological/biological context. We are hesitant to speculate about the ecological function of these taxa as our study explicitly focused on community-level taxonomic trends, not functional profiles, and therefore we sequenced only the ITS and 16S amplicons. We have added additional text to lines 493-495 which acknowledges that future work could explore this question using metatranscriptomics or metagenomics.*

I understand that this is an experimental manuscript, but I am concerned that both the approach of the manuscript and the discussion of the results do not take into account what occurs under natural conditions. Under natural conditions, how does infection occur in this plant species? Or in grasses in general? What are the implications in an ecological context for microorganism communities? Or in an evolutionary context for plant defense against herbivores?

*Author: We agree that ecological context is important for interpreting our results. Thank you for pointing out that our manuscript needed more about this context. In response, we added text to the introduction giving ecological context to the pathogen treatments we chose (lines 80-81) and text comparing these natural modes of transmission to our experimental inoculation methods on lines 175-177 and 182-184. As we selected our methods of experimental inoculation to be similar to each pathogen’s natural mode of transmission, we hesitate to speculate on the implications for microorganism communities or plant defense against herbivores.*

It is not entirely clear why coinfection could modify microbial communities, compared to simple infections. At least, this is not explicitly stated. Including some predictions and hypotheses could help the reader.

*Author: In our discussion, we hypothesize that an altered within-leaf environment due to coinfection could explain changes in fungal communities. We also discuss why this might explain changes in fungal communities, but not bacterial communities (lines 446-448). We have added text to lines 443-444 to explicitly propose potential mechanisms for the changes in diversity we observe in coinfections, but not single infections. Additionally, we have added text to the introduction to provide literature context supporting our hypothesis that coinfections differentially alter microbial communities compared to single infections (lines 64-67).*

Line 68, the idea is unclear.

*Author: The text on line 68 previously read:*

*“their microbes influence each other. Plants can modify their microbial communities”.*

*We appreciate the reviewer’s concern and changed the wording of this sentence to increase clarity (lines 52-56).*

Line 65, the authors attempt to explain the main idea of the manuscript, but it is confusing. Providing some examples of possible scenarios would give the reader more clarity.

*Author: The text on line 65 previously read:*

*“*The interaction between coinfecting pathogens and plant hormones has been”

*We modified the wording of this sentence to increase clarity (line 46).*

Linea 85, It is difficult for me to understand why treatment with salicylic acid is the control treatment.

*Author: We re-worded this sentence to increase clarity (lines 77-80) – salicylic acid is our experimental treatment, and sterile water is our control treatment. We also added clarifying text to lines 89, 139, and 148-149.*

Methods

Line 95, Line 141, What does the control solution contain?

*Author: We have added additional text to lines 78, 89, 139, and 148-149 to clarify earlier that our control solution is sterile water.*

Line 265, Could you describe the characteristics of a symptomatic infection?

*Author: We added text to lines 195-199 to describe the visual indicators of symptomatic infection.*

Reviewer #4

The manuscript titled “Phyllosphere microbial communities are modulated by pathogen coinfection, but not a plant defense hormone” presents interesting findings showing that co-infection by Rhizoctonia solani and Colletotrichum cereale significantly altered and enhanced the diversity of phyllosphere fungal communities in tall fescue, whereas exogenous application of salicylic acid had no significant effect on either fungal or bacterial communities. These results highlight that pathogen interactions play a more dominant role than salicylic acid in shaping phyllosphere microbiomes. Overall, the manuscript is well written and provides valuable insights to the scientific community. However, I recommend several revisions and clarifications before the manuscript can be considered suitable for publication.

*Author: Thank you for taking the time to provide detailed and constructive feedback on our work. We will address the concerns below.*

Line 20-38 Your abstract lacks a clearly defined problem statement and a well-articulated research gap that directly connects to the objectives of your study. In particular, lines 20–24 should emphasize the novelty of your work and how it addresses an existing gap in current knowledge.

*Author: We modified the first sentence of our abstract to more clearly identify the gap in the literature our study aims to fill (lines 20-21).*

The description of treatments involving salicylic acid and plant pathogens (lines 25–27) is somewhat unclear and requires clarification. It is also important to specify the concentration of salicylic acid used in this study. Additionally, please clarify whether you used only a sole inoculation with Rhizoctonia solani or if Colletotrichum cereale was also used in sole inoculation, along with the co-inoculation treatment involving both pathogens. If only R. solani was used for sole inoculation, please justify the rationale for selecting it over C. cereale.

*Author: We modified text on lines 23-27 to clarify our experimental design and co-inoculation treatments and added the salicylic acid concentration to line 24. However, we feel that the rationale for using* R. solani *in single inoculations are details better suited for the Methods section. We note in this section that the low volume of* C. cereale *spores limited inoculations* (lines 166-167) – *our decision to use* R. solani *in single inoculations was driven by practical constraints. Also in our Methods section, we include the rationale for the chosen salicylic acid concentration (lines 141-144).*

In the results section (lines 27–35), the logical flow of presentation needs improvement to support a stronger and more cohesive conclusion. Some statements are also ambiguous — for example, in line 31, the phrase “compared with R. solani, co-inoculation with C. cereale” appears technically and grammatically inconsistent, as it implies co-inoculation only with C. cereale.

*Author: Thank you for pointing out that this line appears inconsistent – as such, we have reworded it (lines 30-31). We believe that this and other suggested rewordings helped to improve the logical flow. To further improve it, we added “In contrast” to the transition from the hormone results to the co-inoculation results.*

Similarly, lines 32–33 are unclear regarding whether sole or co-inoculation had no significant effect on bacterial communities.

*Author: Thank you for this also. We updated the wording of this sentence to clarify that neither inoculation treatment (co- or single) influenced bacterial community structure (line 32).*

Furthermore, the concluding statements of your abstract appear contradictory. You mention that co-inoculation had a stronger influence on fungal communities, yet in the following sentence, you generalize the effect to all microbial communities, despite your results indicating no significant impact on bacterial communities. This inconsistency also extends to the manuscript title, which refers to “phyllosphere microbial communities,” while the results primarily highlight effects on fungal communities.

*Author: We understand how these statements appear contradictory. However, the titles of many recent papers (*From nature to urbanity: exploring phyllosphere microbiome and functional gene responses to the Anthropocene *(Li et al., 2024*)*;* Nutrients and host attributes modulate the abundance and functional traits of phyllosphere microbiome in rice *(Thapa et al., 2017*)*;* Seasonal community succession of the phyllosphere microbiome *(Copeland et al., 2015*)*;* Ecological succession and stochastic variation in the assembly of Arabidopsis thaliana phyllosphere communities *(Maignien et al., 2014) say simply “phyllosphere”, without specifying bacteria or fungi, while only examining one or the other group. In that context, we believe our study meets standards for the usage of “phyllosphere” and “microbial communities” in the current literature. We have added text to the last line of our abstract to highlight our results on fungal communities (line 36).*

Lastly, ensure consistency in terminology—use either “co-infection” or “co-inoculation” throughout the manuscript.

*Author: We appreciate the suggestion for consistency, but feel that using both terms is most appropriate, as “co-inoculation” describes our methodology while “coinfection” discusses ecological outcomes and contextualizes our results within the broader literature.*

It would also strengthen your abstract to include a concise statement on the future research directions or potential implications of your findings, highlighting how they could advance understanding in this field.

*Author: We believe the revised final sentence of our abstract fulfills this suggestion: “Our findings highlight the potential for pathogen coinfections to shape plant-associated microbial communities, particularly fungi, and emphasize the need for further research on the effects of salicylic acid across different host species and experimental approaches.” We think that expanding upon this sentence would be beyond the scope of an abstract.*

Line 38-39 Your manuscript currently lacks a list of keywords relevant to your study. Most journals require keywords to be included immediately after the abstract and before the introduction section. Including well-chosen keywords is important, as they enhance the discoverability and global accessibility of your work within the scientific community. Please add appropriate keywords that accurately reflect the core themes, experimental focus, and major findings of your study.

Author: Per PLOS One’s formatting guidelines, we have not included keywords in the body of the manuscript. Keywords listed on our submission were as follows: phyllosphere, salicylic acid, phytohormone, coinfection, metabarcoding, mycobiome.

Line 40-88 Your introduction lacks a clear and logical flow between statements and paragraphs. For example, in the first paragraph, the discussion shifts from salicylic acid (SA) to general phytohormones and then returns to salicylic acid, which disrupts the coherence of the narrative. To improve the flow, it is recommended to organize the content more systematically—begin by introducing the general role of phytohormones in plant defense mechanisms, and then gradually narrow the focus to salicylic acid and its specific relevance to your study. This progression will help readers follow the rationale of your argument more effectively.

Author: The reviewer makes a good point that the flow of the introduction could be improved. As such, we have re-organized text so that it follows the reviewer’s suggestion: to begin by discussing plant phytohormones, and their general role in the plant immune system, and then to talk about salicylic acid and its relevance in our study.

Additionally, if an abbreviation or acronym is not used later in the text, it should not be introduced, as this can create unnecessary complexity and reduce clarity.

Author: The reviewer is correct that we do not again use the term “systemic acquired resistance (SAR)” making the use of an acronym unnecessary. As such, we have removed it (line 45).

In the final paragraph (lines 76–79), instead of providing details about your experimental setup, it would be more appropriate to focus on developing a clear research gap derived from existing studies. This should highlight why your study is needed and what makes it novel. Based on this identified gap, you can then logically formulate your study objectives and hypotheses to align with the overall research framework. A well-structured introduction should first describe the existing problem or challenge and its impact on plants or microbial communities, followed by a concise summary of the current understanding in the field. It should then clearly define the research gap and lead to the formulation of specific objectives and hypotheses aimed at addressing that gap. Revising your introduction to follow this logical sequence will greatly enhance its clarity, coherence, and scientific strength.

Author: We agree that we could set up our research gap more thoroughly. As such, we have re-organized the introduction and added text to the second to last paragraph of the introduction (lines 70-76). Here, we explain the rationale for our study and identify gaps in the literature.

Line 90-302 It is suggested to reorganize the content of your methodology section to improve clarity and logical flow. For instance, in lines 94–97, you describe the treatments involving salicylic acid and pathogen inoculation, while the details regarding their preparation and application are provided later in the text. This separation makes it difficult for readers to follow. To enhance readability, consider presenting the preparation and application details immediately after the treatment descriptions.

Author: *We appreciate this suggestion, but have retained the current organization of our Methods section, which presents a complete overview of all treatments before describing individual protocols. This structure provides readers with the full experimental context before introducing specific details – to highlight our intent, we have modified text on line 95 to reference Fig. 1 earlier (methodological overview). Starting the Methods section with an overview is also the approach recommended by the recent book,* Scientific Papers Made Easy*, by Stuart West and Lindsay Turnbull, 2023, Oxford University Press.*

Additionally, the concentration of salicylic acid is not mentioned at the point of first reference, which could lead to confusion. Please ensure that the concentration is clearly stated where salicylic acid treatment is first introduced.

Author: Following the recommendation of West and Turnbull (2023; citation above), we have decided that including specifics in this first methods paragraph would not provide a high-level overview as intended. However, we revised the Methods to report the concentration of salicylic acid earlier, where we begin discussing our treatments in detail (line 139), as well as in the abstract (line 24).

Moreover, clarification is needed regarding the limited quantities of C. cereale and the rationale for not using it as a sole inoculation treatment. If the pathogen was available in insufficient quantities, it is unclear why additional inoculum was not prepared for the experiment. Please explain the reasoning behind this decision.

Author: *We appreciate the reviewer's request for clarification. We produce* C. cereale *spores in our lab by growing the fungal culture on agar plates. To avoid contamination, the plates cannot be opened until just before inoculation. Thus, the amount of spores produced can only be determined just before inoculation. Sometimes, for reasons that are not understood, fewer spores are produced than expected. In this experiment, we elected to continue with the inoculation despite the low production of* C. cereale *spores in part because b*oth the experimental plants and fungal cultures take weeks to grow sufficiently and *we did not want to waste the experimentally produced plants or* R. solani *inoculum. Additionally, we were specifically interested in the effects of co-inoculation and estimated that, with our revised experimental design, there would still be enough power to detect that effect. We estimated that if we also retained the* C. cereale *single-inoculation treatment, then we would not have enough replication to detect either effect. Given the logistical constraints, we designed the experiment to make use of available resources.*

Similarly, provide justification for including a mock treatment for sole or co-inoculation when separate sole and co-inoculation treatments were already included.

Author: Separate mock inoculation controls were included for both co-inoculations and single inoculations (lines 89-92). We also added text to lines 92-93 explaining the rationale for using multiple inoculation controls.

In lines 103–108, the discussion of Epichloë coenophiala seems unnecessary within the methodology. Instead, it would be more appropriate to address this background information in the introduction section to provide context for your experimental design.

Author: We agree that our discussion of Epichloë coenophiala was overly extensive – as such, we have removed text from line 108. However, we do not feel that this information fits neatly into our introduction and adding it there would distract from the necessary information, so we limited it to the Methods section.

The seed sterilization protocol should also cite the original reference if it was adopted from previous studies; if it is a modified method, please explain why this particular approach was chosen.

Author: The paper this protocol was adopted from is now cited in line 116.

Furthermore, include complete details of the autoclaving conditions—temperature, pressure, and duration—used for soil sterilization.

Author: *We added the autoclaving conditions to line 121.*

Also, please correct the spelling of “container” in lines 123 and 127.

Author: This spelling is indeed confusing. It refers to a specific product called a “conetainer”. To avoid confusion, we reworded as “conical plastic pots” (lines 122, 124).

The information presented in lines 142–145 would fit better in the introduction section, where it can contribute to building background context.

Author: This information is supplied in the *Methods section where it provides specific context for our chosen salicylic acid concentration. We believe this level of detail is more appropriate for the Methods section than the Introduction, which focuses on broader conceptual context.*

In addition, please specify the exact amount or volume of the 100 mg/L salicylic acid solution applied to each container or plant, and indicate whether it was a single or multiple application during the study period.

Author: We have supplied *a citation in the Methods section for the salicylic acid application protocol we followed (line 144) – within this explanation, we mention that hormone treatments were repeated two days prior to initial treatments. We have also added additional text to lines 140-141 further clarifying our hormone application methodology.*

The number of replications per treatment is also missing. Clarify why 59 plants were used for salicylic acid treatment while 57 plants were used for the control—were the replications unequal across treatments?

Author: Yes. *Plants were randomly assigned to treatments, resulting in 59 plants per treatment group. Unfortunately, two plants from the hormone control treatment did not get moved to growth chambers (experimental error), so they were excluded from the experiment, resulting in uneven numbers of replications per treatment.*

The same for inoculation treatment in lines 168-171.

Author: *Yes, the treatments were unbalanced. The number of co-inoculated plants was limited by the availability of* C. cereale *spores. To avoid loss of statistical power for the effect of inoculating with R. solani alone, we used the available plants for that single-inoculation treatment and its control. Also contributing to these uneven numbers was that the two plants that did not get moved to growth chambers (mentioned above) were both in the mock single inoculation group.*

Lastly, in lines 202–208, if the figure is not included within the text, you may remove the caption and footnote from the main body. Simply cite the figure number in the text and place the corresponding figure, caption, and footnote at the end of the manuscript or in accordance with the journal’s formatting requirements.

Author: *According to the journal's formatting guidelines, figure captions must be placed after the first paragraph where they are cited. Since we recently moved the figure citation earlier in the text to address a previous comment, the caption placement now aligns with journal requirements.*

Line 304-445 It is recommended to make your section headings more concise and descriptive, clearly reflecting the content discussed under each. Avoid using sentence-like headings, as they can reduce clarity and make the manuscript appear less structured. Instead, use brief, focused heading that summarize the main idea or process covered in each section.

*Author: We share the goal of being concise and descriptive. However, we have maintained our current heading structure as it helps readers locate specific results efficiently. We believe this approach enhances readability without compromising manuscript structure. This is also one of the approaches recommended by the recent book,* Scientific Papers Made Easy*, by Stuart West and Lindsay Turnbull, 2023, Oxford University Press.*

Line 447-506 Your discussion section would benefit from a clearer and more logical structure. It is recommended that you first present and interpret your main findings, and then discuss the underlying mechanisms that may explain these results. This should be followed by relevant supporting evidence from previous studies to strengthen your interpretations and demonstrate how your findings align with or differ from existing literature. Such an approach will create a more cohesive and scientifically sound discussion that effectively links your results to established knowledge.

*Author: We thank the reviewer for this feedback – we have implemented it to substantially restructure our discussion.*

Additionally, your manuscript currently lacks a Conclusion section, which is an essential component in most journals. A well-written conclusion should succinctly summarize the key findings of your study in direct relation to your stated objectives and hypotheses. It should also highlight the main take-home messages, identify any limitations of the research, and suggest potential directions for future studies.

*Author: We appreciate this feedback. According to the journal's formatting guidelines, a separate Conclusions section is optional. We believe our Discussion section adequately synthesizes our findings, addresses our research objectives, discusses limitations, and suggests future directions.*

Finally, discussing the practical implications and broader significance of your findings will help demonstrate the contribution of your work to the scientific community and its relevance to future research or practical applications.

*Author: Thank you for this final suggestion. As part of our restructuring of the discussion, we have grouped information on broader significance, and relevance to future research into the last three paragraphs of the discussion. Along with the other suggested revisions, we believe this further improved our manuscript.*

**Final recommendations: Moderate revision**
